# Ovarian tumour growth is characterized by mevalonate pathway gene signature in an orthotopic, syngeneic model of epithelial ovarian cancer

**DOI:** 10.18632/oncotarget.10121

**Published:** 2016-06-17

**Authors:** James B. Greenaway, Carl Virtanen, Kata Osz, Tamas Revay, Daniel Hardy, Trevor Shepherd, Gabriel DiMattia, Jim Petrik

**Affiliations:** ^1^ Department of Biomedical Sciences, University of Guelph, Guelph, ON, N1G 2W1, Canada; ^2^ Princess Margaret Genomics Centre, University Health Network, Toronto, ON, M5G 1L7, Canada; ^3^ Department of Ob/Gyn and Physiology and Pharmacology, Children's Health Research Institute, Western University, London, ON, N6A 5C1, Canada; ^4^ Department of Ob/Gyn and Oncology, Anatomy and Cell Biology, London Regional Cancer Program, Western University, London, ON, N6A 4L6, Canada; ^5^ Department of Biomedical Sciences, University of Guelph, Guelph, ON, N1G 2W1, Canada

**Keywords:** ovarian cancer, mevalonate pathway, simvastatin, p53

## Abstract

Epithelial ovarian cancer (EOC) is the most lethal gynecological cancer and often is not detected until late stages when cancer cells transcoelomically metastasize to the abdomen and typically become resistant to therapy resulting in very low survival rates. We utilize an orthotopic, syngeneic mouse model to study late stage disease and have discovered that the tumor cells within the abdominal ascites are irreversibly re-programmed, with an increased tumorigenicity and resistance to apoptosis. The goal of this study was to characterize the reprogramming that occurred in the aggressive ascites-derived cells (28-2 cells) compared to the original cell line used for tumor induction (ID8 cells). Microarray experiments showed that the majority of genes upregulated in the 28-2 cells belonged to the mevalonate pathway, which is involved in cholesterol biosynthesis, protein prenylation, and activation of small GTPases. Upregulation of mevalonate appeared to be associated with the acquisition of a p53 mutation in the ascites-derived cells. Treatment with simvastatin to inhibit HMG CoA reductase, the rate limiting enzyme of this pathway, induced apoptosis in the 28-2 cell line. Rescue experiments revealed that mevalonate, but not cholesterol, could inhibit the simvastatin-mediated effects. *In vivo*, daily intraperitoneal simvastatin treatment significantly regressed advanced stage disease and induced death of metastatic tumor cells. These data suggest that ovarian cancer cells become reprogrammed, with genetic mutations, and upregulation of the mevalonate pathway, which facilitates the development of advanced stage disease. The use of statins to inhibit HMGCR may provide novel therapeutic opportunities for the treatment of advanced stage EOC.

## INTRODUCTION

Epithelial ovarian cancer (EOC) is the most lethal gynaecologic malignancy and is the fifth leading cause of cancer-related deaths in women [1]. A contributor to the poor prognosis is the late stage of diagnosis, when tumor cells have metastasized to the abdomen and have become more aggressive and resistant to treatment [2]. Using an orthotopic, syngeneic and immunocompetent mouse model of EOC, we have shown that following interaction with the ovarian microenvironment, transformed ovarian epithelial cells derived from ascites (28-2 cells) become irreversibly reprogrammed, with increased proliferative capacity and resistance to apoptosis compared to the original ID8 cell line used for tumor induction [3]. While the source of this reprogramming is still under investigation, interaction with the hormone- and growth factor-rich ovarian microenvironment is implicated. Recent studies have highlighted the importance of the hormonal environment of the ovary, and follicular fluid in particular, that can induce DNA damage and mutations [4].

**Figure 1:**
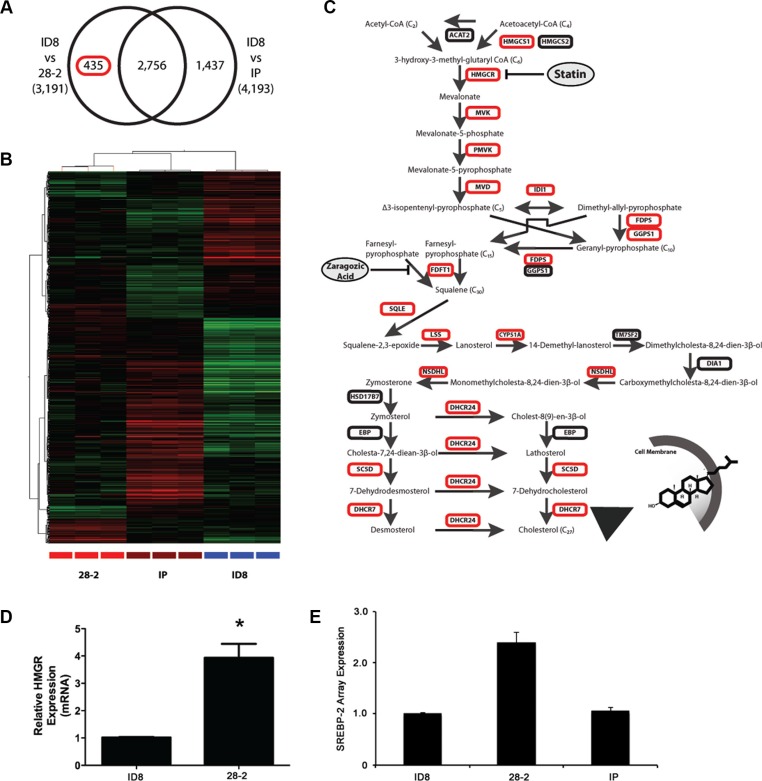
The cholesterol biosynthetic pathway characterizes an ovarian microenvironmental gene signature (28-2) (**A**) Venn diagram illustrating the differential gene expression profiling of mRNA isolated from cells exposed to the ovarian microenvironment (intrabursal - 28-2) or control cells not exposed to the ovarian microenvironment (peritoneum -IP) compared to mouse naive ID8 cells. (**B**) Two-way unsupervised hierarchical clustering of 28-2 specific gene signature indicated in (A), upregulated genes indicated in red and downregulated genes in green. (**C**) Gene ontological analysis of the 28-2 gene signature revealed overrepresentation of cholesterol biosynthetic pathway genes. The cholesterol biosynthetic pathway contains an upstream mevalonate pathway which synthesis isoprenoids, dolichol and ubiquinone. Genes upregulated are indicated in red. (**D**) Relative expression of the rate-limiting enzyme, HMGCR, by quantitative PCR. A significant increase in HMGCR in 28-2 cells validated our expression data *n* = 3 replicates; *p < 0.05. (**E**) Micorarray data showed a 2.4-fold increase in expression of the transcription factor SREBP-2 in 28-2 cells, compared to parental cells used for tumor induction (ID8 cells). There was also an increase in SREBP2 in 28-2 cells compared to cells injected directly into the peritoneum that did not interact with the ovarian microenvironment (IP).

The mevalonate pathway facilitates multiple metabolic functions. The products of the mevalonate pathway include sterol isoprenoids such as cholesterol, and non-sterol isoprenoids such as dolichol, heme-A, isopentenyl, and ubiquinone [5, 6]. In the first committed step of the mevalonate pathway, hydroxymethyl-glutaryl coenzyme A (HMG-CoA) reductase (HMGCR) converts HMG-CoA to mevalonic acid (Figure 1). HMGCR is the rate-limiting enzyme of the mevalonate pathway [6]. Mevalonate can be reduced to isopentenyl pyrophosphate (IPP) and dimethylallyl pyrophosphate (DMAPP). DMAPP is then condensed by farnesyl pyrophosphate (FPP) synthase to form FPP, which is the precursor of cholesterol, steroid, and dolichol biosynthesis. DMAPP can also be condensed by geranylgeranyl pyrophosphate (GGPP) synthase to form GGPP. FPP and GGPP facilitate protein prenylation, which mediates membrane attachment and protein-protein interactions. FPP and GGPP are involved in the prenylation, membrane localization, and activation of small GTPases from the Ras, Rho, Rab and Rac families that regulate a number of important cellular functions and many are well established oncogenes. The Ras-superfamily of proteins is associated with aggressive ovarian cancer cell behaviour and progression of disease (Reviewed in [7]).

Statins, originally designed as lipid-lowering drugs to control hypercholesterolemia, inhibit HMG-CoA reductase activity. This inhibition prevents the formation of cholesterol, but also blocks protein prenylation due to downstream depletion of FPP and GGPP [8]. This upstream inhibition of the mevalonate and cholesterol biosynthetic pathways is important from an anti-cancer perspective [8] and our results suggest that statins may be relevant anti-cancer agents in patients with reprogrammed ascites cancer cells. In a number of cancer cell lines, statins induce apoptosis in a HMG-CoA reductase-specific manner [9], which has intensified interest in the anticancer potential of these drugs.

Epidemiologic data suggests that people on statin therapy may have a reduction in the risk of certain types of cancers, by as much as 50% [10, 11]. In addition to the protective effects of statins, they may also aid cancer treatment by re-sensitizing chemoresistant cancer cells [12]. A proposed mechanism for this re-sensitization is a reduction in the heightened activity of the mevalonate pathway that occurs in cells with *TP53* mutations [13], found in more than half of human cancers [14], and almost universally in high-grade serous ovarian cancer [15]. Combined statin treatment with cytotoxic chemotherapy has resulted in synergistic anti-cancer effects *in vitro* [16–18]. Mutant p53 is known to interact with other transcription factors and modulate expression and function of their target genes [19]. It is noteworthy that *TP53* mutations have been shown to interact with the transcription factors SREBP-2 and nuclear factor Y (NF-Y) [13, 20–23] and the specific R273H oncomorphic mutation associates with SREBP transcription factors to induce upregulation of mevalonate genes [13]. These transcription factors potently induce expression of HMGCR, which catalyzes the formation of mevalonate pathway products [24–26]. Activation of the mevalonate pathway by SREBP-2 has been shown to alter cellular localization and activate YAP and TAZ, which are mediators of the Hippo pathway and are potent oncogenes [27, 28]. Similarly, NF-Y is known to increase expression of the Rho family of small GTPases [29]. Through its interaction with SREBP-2, mutated p53 can influence expression of mevalonate genes [13] and the effects of mutant p53 in breast cancer are mediated through the mevalonate pathway [13].

In ovarian cancer, statins have been shown to induce ovarian cancer cell death and enhance the cytotoxic effects of chemotherapy drugs [30]. *In vitro* combination therapy with cisplatin and fluvastatin synergistically disrupted Ras signalling, resulting in decreased proliferation, and increased apoptosis and cell cycle arrest in EOC cells [31]. Similarly, lovastatin has demonstrated synergistic anti-cancer actions with the chemotherapy drug doxorubicin and can antagonize drug resistance in a host of ovarian cancer cell lines [32]. Although limited data exist on the effect of statins on ovarian cancer progression *in vivo*, the *in vitro* preclinical data suggest that inhibition of the mevalonate pathway may have important therapeutic potential whether this approach is used alone or in combination with traditional cytotoxic chemotherapy.

We hypothesized that inhibition of the mevalonate pathway would reduce tumor cell viability *in vitro* and inhibit tumorigenicity and metastatic potential in an *in vivo* mouse model of advanced stage EOC.

## RESULTS

### Exposure to the ovarian microenvironment upregulates the mevalonate pathway in murine ovarian cancer cells

An orthotopic, syngeneic mouse model of epithelial ovarian cancer (EOC) was used in which cells were injected under the ovarian bursa which allows the tumor cells to colonize, invade through the basement membrane and gain exposure to the ovarian microenvironment. Previous work in our lab has shown that following interaction with the ovarian microenvironment, these cells have accelerated tumor growth and increased morbidity (3). Our work shows that cell lines established after exposure to the ovarian microenvironment (28-2 cells) demonstrate an accelerated mitotic index, increased protein expression of angiogenic, survival and proliferative proteins, augmented migratory capacity and form tumors more rapidly than cells exposed directly to the peritoneal microenvironment via intraperitoneal (IP) injection (IP cells) [33]. Unsupervised clustering of the filtered set of 33235 probes revealed that each individual line had a distinct molecular profile. To identify genes that may be responsible for the increased tumorigenicity observed in cells exposed to the ovarian microenvironment, we profiled 28-2, IP and ID8 cell lines on Agilent Whole Mouse Genome arrays. Analysis of gene expression by ANOVA *(p* < 0.05) with a Tukey's *post hoc* test identified the following differentially expressed probes: 2,756 probes significantly different between 28-2 and ID8 lines; 4193 probes significantly different between the ID8 and lines derived after IP injection; and 3191 probes significantly different between the 28-2 and IP-injection (Figure 1). A 28-2 specific gene list was generated by analyzing the overlapping set of probes common between the two 28-2 comparisons (28-2 vs IP; 28-2 vs ID8), yielding 435 probes which are specific to cells reprogrammed after exposure to the ovarian microenvironment. Two-way hierarchical clustering illustrates a unique separation of transcripts and their abundance between the 28-2 cells and the other cell lines (Figure 1B). Gene ontological analysis of the 28-2 cell gene expression signature revealed significant enrichment in a number of categories belonging to the ‘cholesterol biosynthetic process’, ‘cholesterol metabolic process’, ‘steroid biosynthetic process’ and ‘lipid metabolic process’ (*p* < 0.001) gene ontologies, amongst other biological processes (Table 1). To explore this further, we looked specifically at the cholesterol biosynthetic pathway (Figure 1C), which converts the two-carbon metabolite acetyl-CoA into the 27-carbon cholesterol molecule through a series of enzymatic steps. Members of the cholesterol biosynthetic pathway up-regulated in the 28-2 gene signature are indicated by a red box in Figure 1C. The rate-limiting step of the pathway occurs upstream with the conversion of 3-hydroxy-3-methyl-glutarylCoA into mevalonate by 3β-hydroxy3-methylglutharyl coenzyme A reductase (HMGCR). Downstream of HMGCR is the mevalonate pathway comprised of lipids that are synthesized from the 5-carbon precursors, isopentenyl-pyrophosphates, into the 15-carbon farnesyl pyrophosphate (FPP) and the 20-carbon geranylgeranyl pyrophosphates (GGPP). Both FPP and GGPP are enzymatically fused to the C-terminus of proteins through a process called prenylation, which provides a lipid attachment for small GTPases such as Ras, Rac, Rho and CDC42. Fusion of two FPP metabolites by squalene synthase is the first committed step to cholesterol synthesis and is mediated by squalene synthase. Further modifications of squalene form cholesterol, which functions in maintaining plasma membrane fluidity and is a precursor for steroid hormone synthesis. As shown in Figure 1, genes involved in all aspects of cholesterol biosynthesis, farnesylation, geranylation, and protein prenylation are expressed significantly higher in the 28-2 cell population compared to the other populations. We also queried the array for expression of SREBP-2, which transcriptionally activates the mevalonate pathway and found that 28-2 cells show a 2.4-fold increase in expression compared to ID8 cells or cells collected following intraperitoneal injection (Figure 1E).

**Table 1:** Increased expression of genes in the 28-2 gene signature

GO ACCESSION	GO Term	corrected *p*-value	Count in Selection
GO:0009058	biosynthetic process	0.0934628	80
GO:0003824	catalytic activity	0.002051266	130
GO:0044255	cellular lipid metabolic process	0.0934628	21
GO:0044237	cellular metabolic process	0.048637476	143
GO:0006695	cholesterol biosynthetic process	7.618883E-9	11
GO:0008203	cholesterol metabolic process	5.409889E-4	11
GO:0005622	intracellular	0.001581868	227
GO:0043231	intracellular membrane-bounded organelle	0.001581868	177
GO:0043229	intracellular organelle	0.001243249	196
GO:0044424	intracellular part	0.001805784	222
GO:0009240	isopentenyl diphosphate biosynthetic process	0.008654	3
GO:0019287	isopentenyl diphosphate biosynthetic process, mevalonate pathway	0.008654	3
GO:0046490	isopentenyl diphosphate metabolic process	0.008654	3
GO:0008299|GO:0009241	isoprenoid biosynthetic process	0.00287574	6
GO:0008610	lipid biosynthetic process	0.001243249	19
GO:0006629	lipid metabolic process	0.086196415	27
GO:0030324	lung development	0.010353317	11
GO:0043227	membrane-bounded organelle	0.001581868	177
GO:0008152	metabolic process	5.409889E-4	188
GO:0043226	organelle	0.001189004	197
GO:0008654	phospholipid biosynthetic process	0.009288681	10
GO:0030838	positive regulation of actin filament polymerization	0.09294873	4
GO:0044238	primary metabolic process	0.085513584	143
GO:0060541	respiratory system development	0.024802865	11
GO:0030323	respiratory tube development	0.011691348	11
GO:0006694	steroid biosynthetic process	5.409889E-4	11
GO:0016126	sterol biosynthetic process	6.5381165E-8	11
GO:0016125	sterol metabolic process	0.00117981	11
GO:0016740	transferase activity	0.057958957	52

### Inhibition of HMGCR by simvastatin induces apoptosis *in vitro*

**Figure 2:**
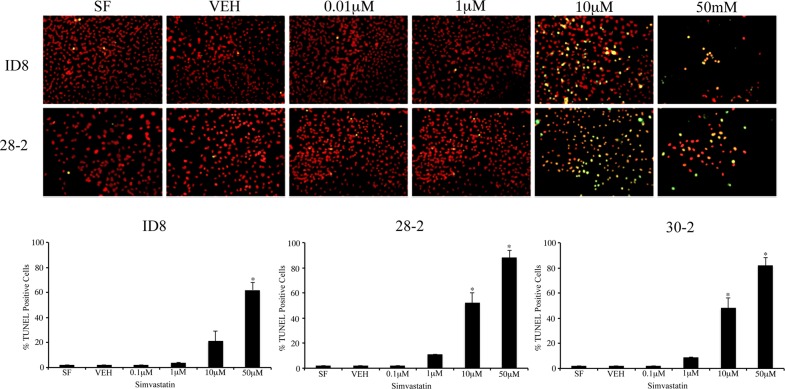
Inhibition of HMG-CoA induces apoptosis of MOSEC *in vitro* (**A**) Simvastatin treatment of ID8 and 28-2 cells induce apoptotic cell death measured by the TUNEL assay in a dose-dependent manner. TUNEL positive cells (yellow) exhibit characteristic pyknotic morphology characterizing apoptotic cells. Cells were fixed on coverslips 24 hrs after simvastatin treatment. (**B**) Analysis of percentage of TUNEL positive cells reveals a significant (*p < 0.001) increase in higher doses of simvastatin when compared to controls (SF). *N* = 3, one-way ANOVA, Dunette's poc hoc test. SF - serum free control, VEH - simvastatin vehicle control, 0.1 μM – 0.1 μM dose of simvastatin, TUNEL - terminal deoxynucleotidyl transferase dUTP nick end labeling.

To interrogate the functional relevance of the cholesterol biosynthetic pathway upregulation in our model, we treated the original ID8 cells and two ascites-derived cell lines, 28-2 and 30-2,with simvastatin to inhibit the rate-limiting step of cholesterol and mevalonate synthesis and measure specific *in vitro* effects. We observed a dose-dependent decrease in cell viability after 96-hour simvastatin administration with an IC_50_ of 11.5 μM in the native ID8 cells, 3.9 μM in 28-2 cells and 5.8 μM in 30-2 cells. We extended our findings to include a variety of human cell lines including EOC cell lines (SKOV3 and OVCAR3) and normal ovarian surface epithelial cells (NOSE). Simvastatin decreased viability in all cell lines tested and the IC_50_ values were determined (Table 2). To confirm the decrease in viability was due to cell death, ID8 and 28-2 cells were grown on coverslips and apoptosis was quantified using the TUNEL assay after simvastatin treatment (Figure 2). TUNEL positive, pyknotic nuclei were first observed in the 10 μM simvastatin group and high rates of apoptosis were recorded in the 50 μM simvastatin treatment groups in accordance with our viability data. At the 10 μM simvastatin dose, 28-2 and 30-2 cells showed a significant (*p* < 0.001) increase in apoptosis compared to untreated controls, whereas at the 50 μM dose all three cell lines (ID8, 28-2 and 30-2) had significantly higher (*p* < 0.001) apoptotic cell death (Figure 2).

**Table 2:** Simvastatin IC_50_ values determined from WST1 assay

Cell Line	IC_50_
ID8	11.5 μM
28-2	3.9 μM
30-2	5.8 μM
SKOV3	13.8 μM
OVCAR3	7.2 μM
NOSE	3.7 μM
iOvCa130 p53 WT	10.2 μM
iOvCa147 p53 MUT	2.9 μM

### Mevalonate, not cholesterol, mediates simvastatin-induced cell death

**Figure 3:**
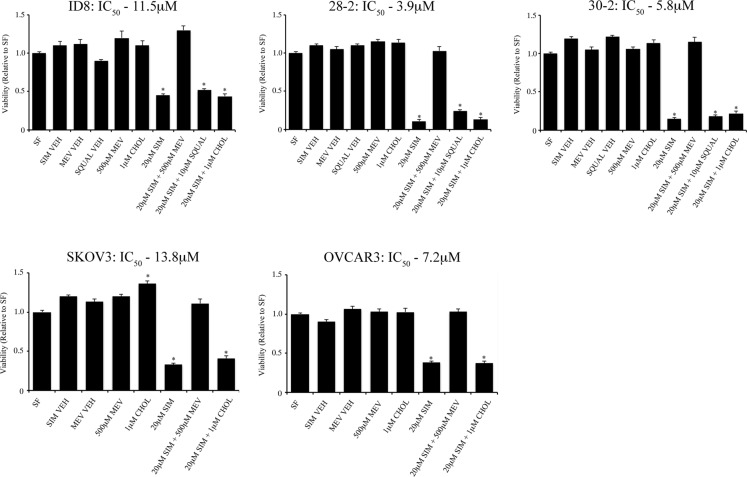
Mevalonic acid, but not cholesterol can rescue EOC cell lines from simvastatin induced cell death Treatment of 500 μM mevalonic acid (MEV) rescues various mouse (ID8, 28-2, 30-2) and human (SKOV3, OVCAR3) cell lines from 20 μM simvastatin cell death determined by viability assay. Administration of water-soluble cholesterol (CHOL) did not rescue all cell lines tested from simvastatin induced cell death. SF - serum free, NOSE - normal ovarian surface epithelial cells, SIM VEH - simvastatin vehicle control, MEV VEH - mevalonic acid vehicle control, SQUAL - squalene.

In order to specifically identify the role of the cholesterol and mevalonate biosynthetic pathways in maintenance of viability in the mouse EOC lines, we treated cell lines with simvastatin and performed rescue experiments by adding back mevalonate and cholesterol (Figure 3). Administration of 500 μM mevalonate rescued all cell lines tested (ID8, 28-2, 30-2, SKOV3, OVCAR3 and NOSE) whereas cholesterol could not rescue at any of the doses tested (0.01, 0.1, 1.0, 10, 100 μM – data not shown). Treatment of MOSEC with 0.01 μM – 50 μM squalene could not rescue cells from simvastatin induced cell death (data not shown); thus, we concluded that metabolites downstream of mevalonate and upstream of squalene are critical for survival of these cells after HMGCR inhibition.

### Geranylgeranyl pyrophosphate rescues MOSEC from simvastatin-induced apoptosis

**Figure 4:**
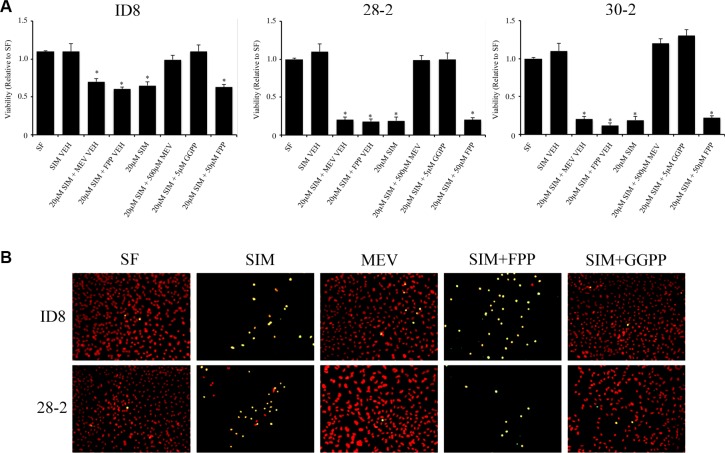
Geranylgeranyl pyrophosphate (GGPP), but not farnesyl pyrophosphate (FPP) rescues mouse ovarian cancer cell lines from statin induced cell death (**A**) Transformed MOSEC lines co-treated with simvastatin and GGPP, prevented simvastatin induced cell death measured by WST-1 viability assay. Interestingly, coadministration of FPP did not rescue MOSECs cells after HMGCoA inhibition by simvastatin. Viability was measured by WST1 assay. *p < 0.05 (**B**) TUNEL analysis of simvastatin, mevalonic acid, GGPP and FPP treated MOSEC lines confirm that the downstream metabolites, mevalonic acid and GGPP, can rescue MOSEC lines from simvastatin induced apoptosis. FPP was unable to rescue simvastatin induced cell death. SF - serum free control, SIM VEH - simvastatin vehicle control, SIM - simvastatin, MEV VEH - mevalonic acid vehicle, FPP VEH - farnesyl pyrophosphate vehicle control, GGPP - geranylgeranyl pyrophosphate.

The mevalonate pathway functions to generate various 5-carbon isopentyl-pyrophosphates building blocks to form isoprenoids, including FPP, GPP and GGPP. As isoprenoids are critical determinants of small GTPase activity by directing proper subcellular localization to the plasma membrane, we hypothesized that inhibition of HMGCR by simvastatin may deplete the cellular pool of FPP or GGPP and prevent proper prenylation of small GTPases, reducing their activity, leading to apoptosis. Following co-treatment of MOSEC cells with 20 μM simvastatin and 5 μM GGPP, GGPP reversed the simvastatin-induced apoptotic cell death in MOSEC cells (Figure 4). Co-treatment with 50 μM FPP did not elicit the same rescue effect and a significant decrease in viability was observed (Figure 4).

### Simvastatin inhibits tumor growth in the syngeneic model and reduces cell migration *in vitro*

**Figure 5:**
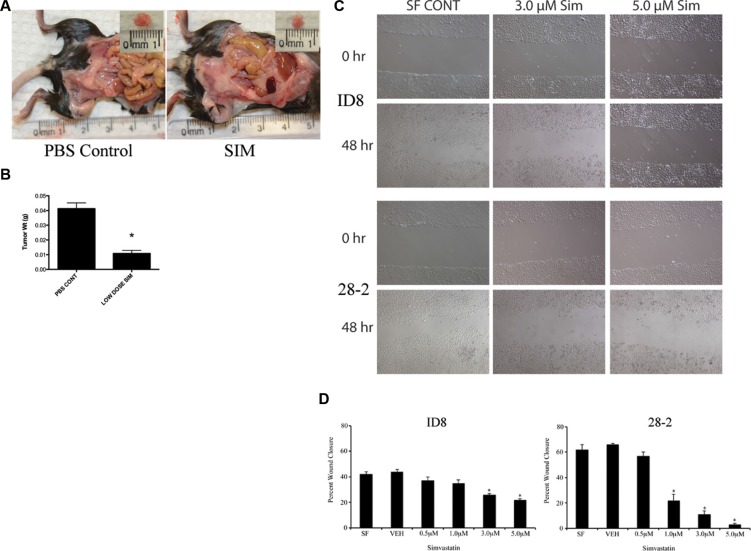
Simvastatin treatment reduces scratch wound healing *in vitro* and tumor weight *in vivo* (**A**–**B**) 1.0 × 10^6^ ID8 cells were placed in the ovarian bursa and permitted to recover for a week before receiving 1 mg/kg/day simvastatin IP for 60 days. At necropsy, SIM treated mice had smaller tumors with decreased weight *p < 0.05. (**C**) Representative photomicrographs of simvastatin treated ID8 and 28-2 cells at 0 and 48 hours timepoints after scratch wounding. (**D**) Quantification of the percent scratch wound closure reveals a significant (*p < 0.05) decrease in closure at higher doses of simvastatin. PBS CONT - PBS injected control, SIM - 1 mg/kg/day simvastatin treated group, IP - intraperitoneal.

**Figure 6:**
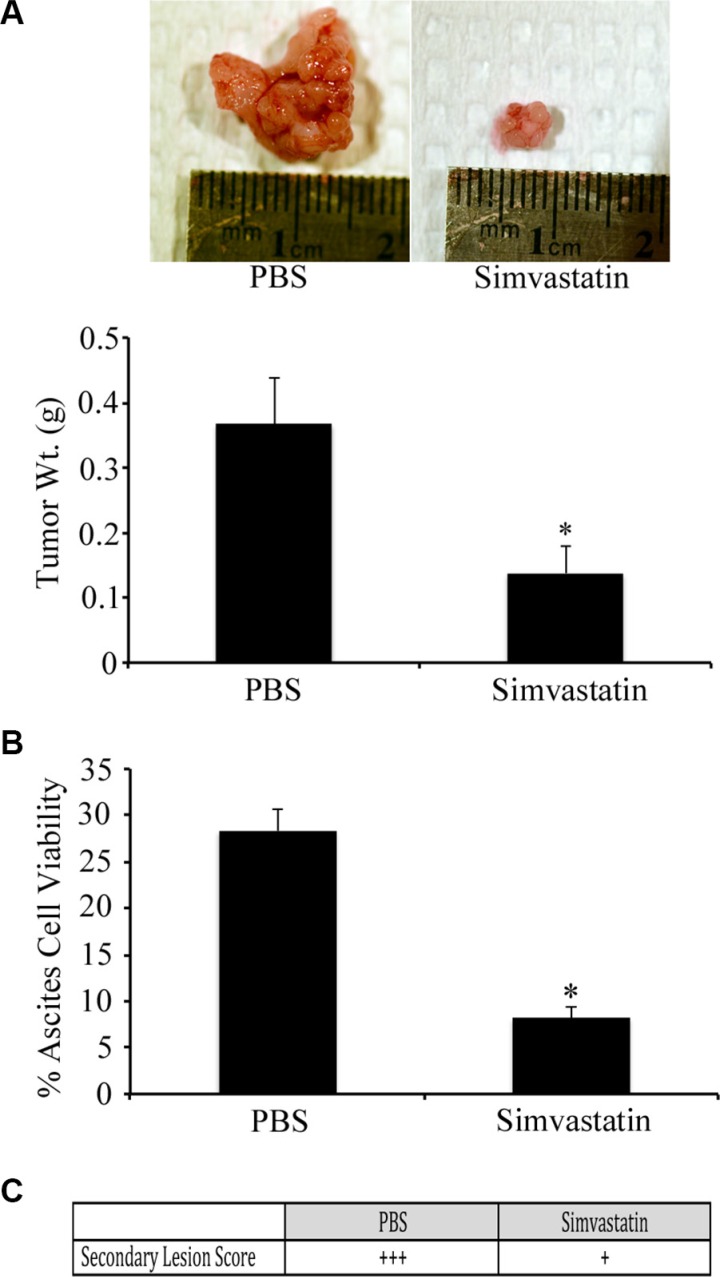
Simvastatin induces regression of advanced stage ovarian cancer Tumors were orthotopically induced with ID8 cells in syngeneic mice and allowed to grow until 60 d PTI at which time they received daily IP injections of PBS or simvastatin (1 mg/kg/d) for 20 d. (**A**) Tumors from mice treated with simvastatin had regressed to a significantly (*p < 0.05) smaller size, compared to PBS treated controls. (**B**) Simvastatin treated mice had a significant (*p < 0.05) reduction in percentage of viable ovarian cancer cells collected from ascites. (**C**) Simvastatin treatment reduced the number of metastatic peritoneal tumors, compared to PBS treated controls.

To investigate the *in vivo* effects of HMG inhibition in EOC, we applied our syngeneic, orthotopic mouse model of EOC in which spontaneously-transformed murine ovarian surface epithelial ID8 cells were injected into the bursa of recipient mice. Following tumor induction, mice received daily intraperitoneal injections of simvastatin (1 mg/kg/day) or PBS. Mice were sacrificed on day-60 and tissues were measured and collected. Mice that received daily IP injections of simvastatin demonstrated a significant reduction in tumor size and weight when compared to tumors collected from the PBS injected control group (Figure 5; *p* < 0.05). Murine EOC cells were also treated with sublethal doses of simvastatin for 48hrs, and a scratch assay was performed to identify the effects on cell migration. The migration of 28-2 cells was inhibited to a greater extent than ID8 cells (Figure 5C–5D). Simvastatin treatment was also able to reduce disease morbidity in advanced-stage ovarian cancer. When treatment was initiated at 60 days post tumor induction (PTI), there was significant (*p* < 0.05) tumor regression (Figure 6A). There was also a reduction (*p* < 0.05) in the viability of cells collected from ascites from simvastatin treated mice (Figure 6B). and reduced number of metastatic peritoneal tumors (Figure 6C).

### Ascites-derived cells following orthotopic tumor initiation are p53 mutant and have genomic instability

**Figure 7:**
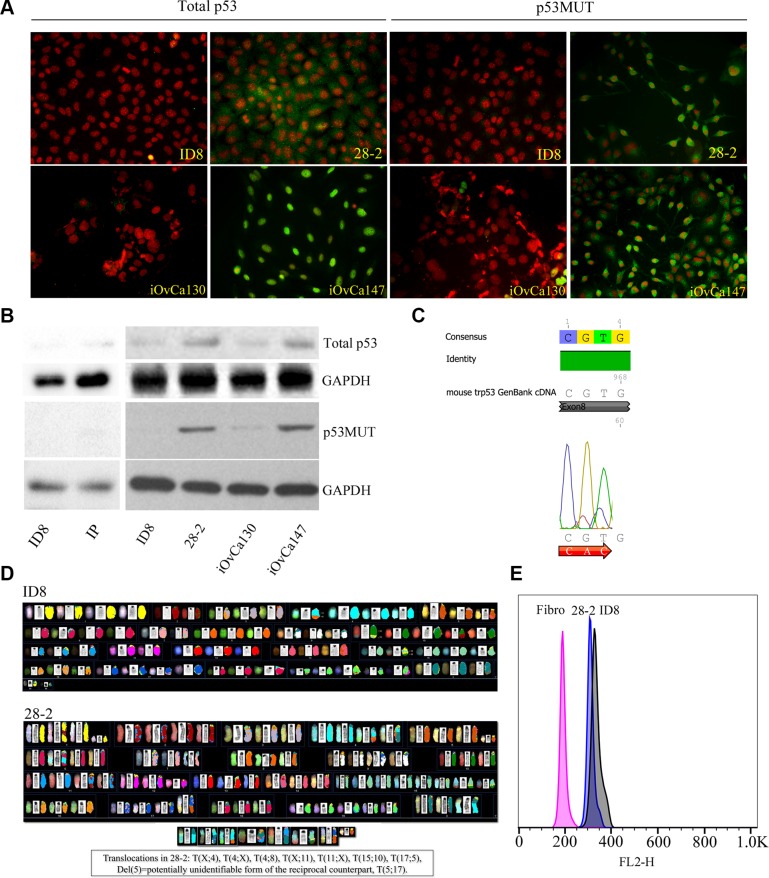
Reprogrammed EOC cells exhibit p53 mutations and genomic instability (**A**) P53 MUT human EOC cells (iOvCa147, validated to have p53 mutation in Exon 6 – sequencing performed by Dr. G. DiMattia) showed increased expression of p53 protein compared to p53WT cells (iOvCa130; verified p53 WT – sequencing performed by Dr. G. DiMattia) using immunofluorescence with specific antibodies for each isoform. Similarly, reprogrammed murine ascites-derived EOC cells (28-2) had increased expression of WT p53 and mutant p53 protein, compared to the non-reprogrammed parental murine ID8 EOC cells. (**B**) Cell lysates were subjected to Western blot analysis for expression of total and mutant p53. 28-2 and iOVca147 cells demonstrated increased expression of total and mutant p53 compared to ID8, IP, and iOvCa130 cells. (**C**) 28-2 cells have a CGT to CAT mutation in Exon 8 of the p53 gene. (**D**) Spectral karyotyping showed extensive numerical and structural chromosome abnormalities in both ID8 and 28-2 cells. The presence of three translocations and three deletions specific to 28-2 emphasizes the activity of structural rearrangements during its development and adaptation. (**E**) The highly amplified genomes of 28-2 and ID8 cells were visualized by the G1 peaks of the cell cycle as measured by flow cytometry and compared to a diploid fibroblast control. Numerical instability resulted in a ~6.2% smaller genome size of 28-2, as visible from the difference between the G1 peaks of 28-2 vs. ID8 cells.

We hypothesized that the more aggressive phenotype of the 28-2 cells may be related to the presence of mutated *TP53*, which is seen in the majority of high-grade serous ovarian cancers in humans. Human ascites-derived cells known to be *TP53* wild-type (iOvCa130) and *TP53* mutant (iOvCa147) as well as murine ID8 and ascites-derived 28-2 cells were subjected to immunofluorescence and Western blot analysis with antibodies specific for total p53 and mutant p53. iOvCa147 and 28-2 cells exhibited high expression of total and mutant p53, compared to iOvCa130 and ID8 cells (Figure 7A–7B). Sequence analysis of 28-2 cell p53 cDNA detected a mutation in codon 273 (CGT-CAC;Arg-His) of exon 8 (Figure 7C).

Chromosomal analysis revealed significant genomic instability with severe numerical and structural abnormalities in both ID8 and 28-2 cell lines (Figure 7D). The ID8 genome is more than trisomic, being mostly tetrasomy for Chr 1, 2, 5, 8, 10, 17 and five copies of Chr 6, 15 and 19. Centric fusions were not observed, however all metaphases contained two reciprocal translocations der(4)T(4;8) and der(15)T(10;15). The derivative Chr 8 and Chr 10 counterparts were only observed in 1 metaphase. These results partially confirm the G-banded karyotype described by Roby et al. [34], specifically the presence of trisomies and tetrasomies, although with some differences. We could not detect the T(15;18) suggested by Roby et al. [34], but identified two other reciprocal translocations. The 28-2 karyotype is clearly derived from the ID8 genome, showing very similar chromosomal abnormalities with some differences: less copies of Chr 6, 8, 10, X, but tetrasomy of Chr 4, 11 and 6-8 copies of Chr 15 including mostly two copies of the der(15)T(10;15) translocation. The other translocation from ID8 der(4)T(4;8) was also present in every metaphase. Three unique reciprocal translocations were identified in 28-2, including T(4;X), T(5;17) and T(11;X). Both derivative counterparts of these rearrangements were observed, although der(5)T(5;17) was mostly present as del(5). Frequent deletion of one copy of Chr 1 and 12 is also specific to 28-2. Centromere amplification or dicentric chromosomes were also observed in both cell lines, but much more frequently in 28-2 as compared to ID8 (41% vs 20%, respectively). Flow cytometric comparison of G1 peaks of the cell cycle confirmed the highly amplified genomes in both ID8 and 28-2, as compared to the normal fibroblast control (327, 308, 190, respectively), but also showed a slight reduction (6.2%) of genome size in 28-2 compared to ID8 (Figure 7E).

**Figure 8:**
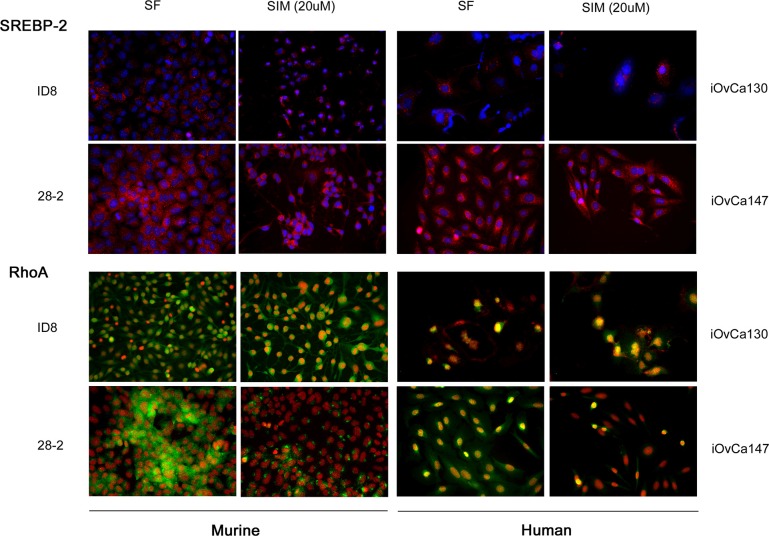
p53MUT (28-2, murine; iOvCa147, human) have increased SREBP-2 and RhoA expression compared to p53WT (ID8, murine; iOvCa130, human) EOC cells Increased expression of the HMGCR transcription factor SREBP2 was seen in p53MUT compared to p53WT cells (SREBP2 red, nuclei blue). Expression of the small GTPase RhoA, which is a downstream protein in the mevalonate pathway was upregulated in the p53MUT ascites derived cells (RhoA green, nuclei red). Treatment with the mevalonate pathway inhibitor simvastatin (20 μM) caused greater inhibition of RhoA expression in p53MUT metastatic ascites-derived cells (28-2, iOvCa147) than primary tumor cells (ID8) or p53WT (iOvCa130) ascites derived cells.

**Figure 9:**
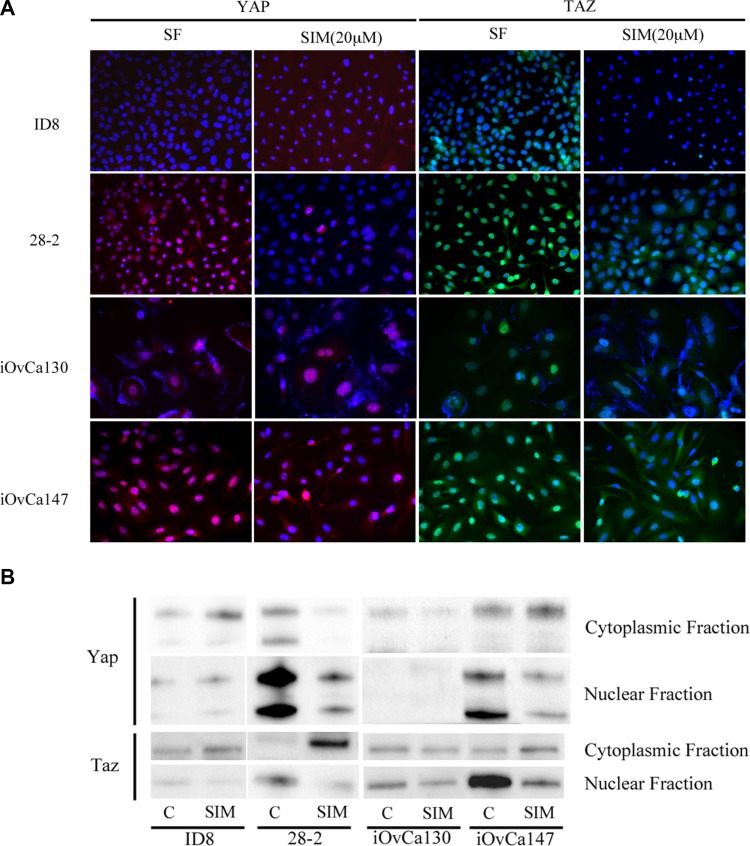
Simvastatin induces change in YAP and TAZ expression from nuclear to cytoplasmic localization in p53MUT EOC cells (**A**) P53MUT 28-2 (murine) and human (iOvCa147) ascites-derived EOC cells show strong nuclear localization of YAP (red) and TAZ (green), while p53WT murine (ID8) and human (iOvCa130) EOC cells exhibit reduced or cytoplasmic staining. Treatment with 20 μM Simvastatin for 24 h resulted in reduced expression of YAP and TAZ and more cytoplasmic localization. Nuclei are counterstained blue with DAPI stain. (**B**) Cells were lysed and separated into cytoplasmic and nuclear fractions and subjected to Western blot analysis of Yap and Taz. Simvastatin treatment resulted in a shift in expression of YAP and Taz from nuclear to cytoplasmic localization and this shift was more pronounced in 28-2 and iOvCa147 cells. ID8 and 28-2 lanes were run and imaged on the same gel but are shown separately as some extraneous lanes were omitted.

**Figure 10:**
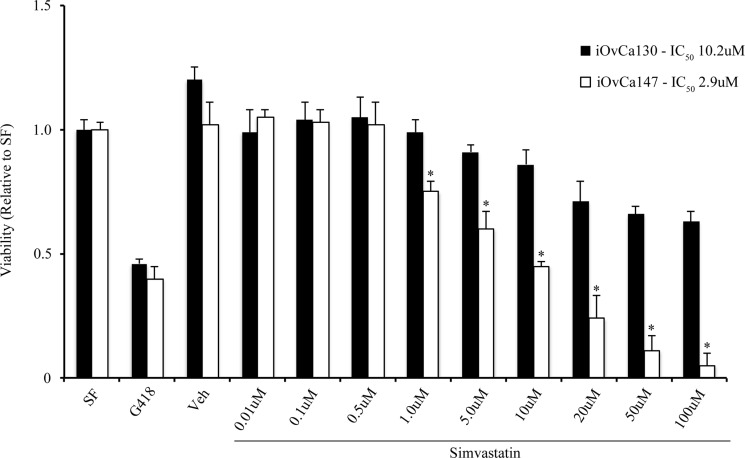
Simvastatin reduces viability of human ascites-derived EOC cells P53WT (iOvCa130) and p53MUT (iOvCa147) cells were cultured in the presence of Simvastatin (20 μM) for 24 hr and subjected to WST-1 assay to measure viability. EC_50_ values were calculated for cell response to simvastatin for each cell type. Simvastatin had a more potent effect on viability in iOvCa147, compared to iOvCa130 cells. *indicates statistical difference between cell lines for each treatment concentration (*p* < 0.05); *n* = 3 replicates.

iOvCa147 and 28-2 cells exhibited increased expression of SREBP-2 and the small GTPase RhoA (Figure 8). Simvastatin treatment did not alter expression of SREBP-2, but did result in a decrease in expression of RhoA in 28-2 and iOvCa147 cells, but not iOvCa130 or ID8 cells (Figure 8). Immunofluorescence was also performed for the members of the HIPPO pathway YAP and TAZ as this pathway has been implicated in cancer onset and progression and YAP and TAZ are activated by small GTPases such as RHO [28]. 28-2 and iOvCa147 cells had increased staining for these factors, with prominent nuclear localization, compared to ID8 or iOvCa130 cells (Figure 9). Simvastatin treatment reduced expression of YAP and TAZ and induced a shift to cytoplasmic localization in 28-2 and iOvCa147 cells (Figure 9). Cell fractionation experiments confirmed a shift from nuclear to cytoplasmic localization of Yap and Taz following simvastatin treatment, particularly in the p53 mutant 28-2 and iOvCa147 cells (Figure 9). Human ascites derived ovarian cancer cells known to be wild-type for *TP53* (iOvCa130) and *TP53* mutant (iOvCa147) were treated with increasing doses of simvastatin for 24 h and subjected to WST-1 assay to quantify cell viability. iOvCa147 cells had enhanced sensitivity to simvastatin, compared to iOvCa130 cells, with a significant (*p* < 0.05) reduction in viability at all doses beyond 0.5 μM (Figure 10).

### Ascites-derived cells have increased H3 acetylation at the HMGCR NF-Y binding site

**Figure 11:**
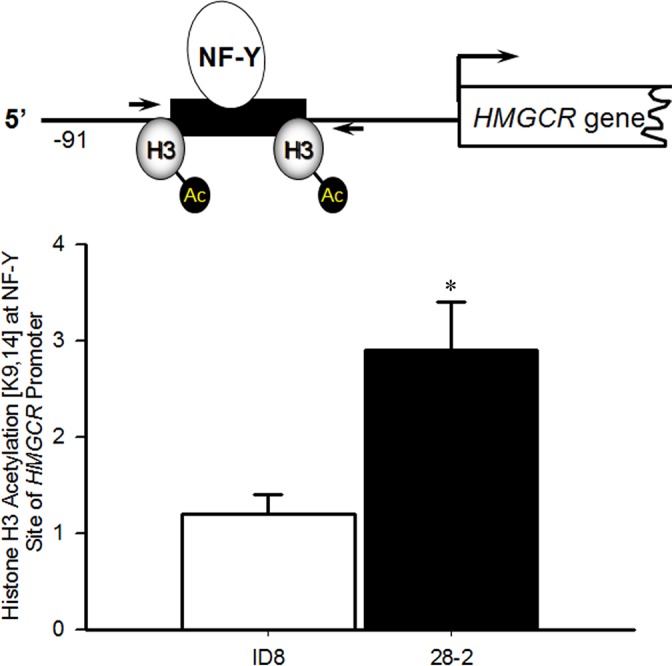
Increased acetylation of histone H3 [K9,14] surrounding the NF-Y site in the promoter of HMGCR in Re-programmed 28-2 Cells ChIP analysis (*n* = 3 independent samples) demonstrated increased acetylation of the NF-Y site in the promoter of HMGCR in the ascites-derived 28-2 cells compared to the parental ID8 cells used for tumor induction. *p < 0.05 by *t*-test.

It is well established that increases in the acetylation of histone H3 at K9 and K14 is associated with chromatin activation and increased RNA polymerase II recruitment [35, 36]. Therefore to investigate if transformation of these cells led to augmented HMGR transcription as a result of increased histone H3 [K9,14] acetylation, chromatin immunoprecipitation (ChIP) was employed in ID8 and 28-2 cells. ChIP revealed increased acetylation of Histone H3 [K9/K14] within the NF-Y site (−68 to −64) of the HMGCR promoter in 28-2 cells, compared to ID8 cells (Figure 11), suggesting epigenetic activation of HMGCR. The non-specific binding of IgG was tested and found to be minimal (Ct value > 34, data not shown).

### Metastatic peritoneal tumors have greater expression of members of the mevalonate pathway compared to primary tumors

**Figure 12:**
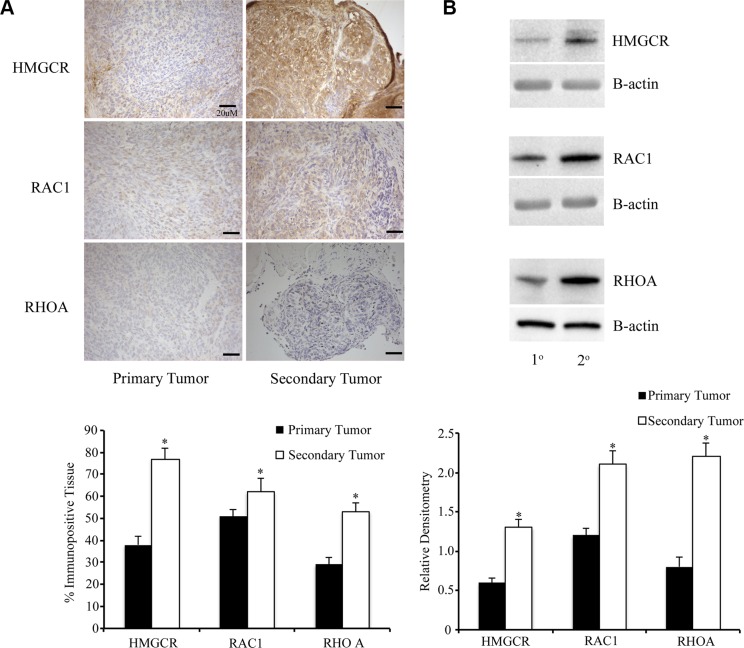
Metastatic peritoneal tumors have increased expression of members of the mevalonate pathway (**A**) Primary and metastatic tumors from ID8 injected mice (*n* = 6/group) were collected and stained for HMGCR and small GTPases Rac1 and RhoA. Metastatic secondary tumors had a significantly (*p < 0.05) higher percentage of tissue immunopositive for the enzyme and GTPases than primary tumors. Scale bars are 20 μM (**B**) Western blot was performed on tumor lysates from primary and metastatic tumors. Metastatic tumors had higher expression of HMGCR, Rac1 and RhoA, compared to primary tumors (*p < 0.05).

At 90d PTI, primary tumors and metastatic peritoneal tumors were collected, fixed, and subjected to immunohistochemistry or Western blot analysis for HMGCR and the small downstream GTPases Rac1 and RhoA. Following IHC, software analysis was used to quantify the percentage of immunopositive tissue in both the primary ovarian tumors as well as the metastatic peritoneal tumors. There was increased expression of HMGCR and both GTPases in the metastatic peritoneal tumor tissue, compared to the primary tumors. Although the metastatic tumors were smaller, they had increased levels of all three proteins (Figure 12A–12B).

## DISCUSSION

We have demonstrated that MOSEC lines exposed to the ovarian microenvironment increase expression of various enzymes of the mevalonate pathway. Our data also show that isoprenoid generation is critical for the viability of these cell lines and that simvastatin can inhibit the growth of ovarian lesions in our immunocompetent mouse model of EOC. We have previously characterized the effects of injecting ID8 cells orthotopically into the ovarian bursa of recipient mice and compared the effects to mice that receive ID8 cells injected directly into the peritoneal cavity and discovered a striking reprogramming and aggressiveness following interaction with the ovarian microenvironment [3]. Data from the current study demonstrate that exposure to the ovarian microenvironment significantly increases expression of members of the mevalonate pathway.

To assess the effect on cell viability, we utilized statins to inhibit the mevalonate pathway. Statins are potent inhibitors of HMG-CoA reductase, which catalyze formation of mevalonate from HMG-CoA and is the rate-limiting step in the mevalonate and cholesterol biosynthetic pathways [6]. *In vitro* treatment of MOSEC lines with simvastatin revealed it induced apoptosis in a dose-dependent manner. Interestingly, the ID8 parental cell line was less sensitive to simvastatin-induced apoptosis when compared to the 28-2 and 30-2 cell lines, which were derived from ascites following interaction with the ovarian microenvironment. Concurrent treatment of MOSEC lines with simvastatin and mevalonate prevented simvastatin-induced apoptosis whereas squalene and cholesterol could not rescue MOSEC lines. We concluded that a metabolite generated in mevalonate pathway, which is upstream of squalene and cholesterol, must be essential for the viability of MOSEC lines. Studies have shown that statins can induce apoptosis through activation of the p38 [37], caspase-3 [38], DR5/CHOP/pJNK [39], Bax [40], TRAIL [41] and suppression of Akt [38, 42], Bcl-2 [40, 43], mTOR [42]. Studies in *C. elegans*, suggest depleting the cells of isoprenoid units initiates the endoplasmic reticulum unfolded protein response (UPR^er^) followed by growth arrest and apoptosis [44–46]. Current investigations in our laboratory are underway to identify the pathway(s) that mediate simvastatin induced apoptosis.

The mevalonate pathway includes the formation of various isopentenyl pyrophosphate base molecules including farnesyl-pyrophosphate (FPP) and geranylgeranyl-pyrophosphate (GGPP) downstream of mevalonate synthesis. Collectively FPP, GGPP and other isopentenyl-pyrophosphate molecules (termed “isoprenoids”) take part in post-translational modification of proteins through a process termed prenylation [45–47]. Target proteins like the Ras superfamily of small GTPases require prenylation for proper function by localizing them to the plasma membrane in close proximity to their substrates [48]. It has been predicted that over 300 proteins undergo prenylation including Ras, Rho, RAB and ARF [49]. Due to the observation that statin-induced cell death could be rescued by mevalonate, but not squalene or cholesterol, we tested whether isoprenoids were required to prevent statin-induced apoptosis. We identified GGPP as the isoprenoid required to rescue MOSEC cells from apoptotic cell death. Prenylation of target proteins with GGPP, or geranylation, occurs with Rho, RAB and ARF, implicating a loss of subcellular localization and thus function leading to cell death in our model.

Our study also illustrated that daily treatment of 1 mg/kg simvastatin *in vivo* resulted in a significant decrease in tumor progression at 60 days post tumor induction. Retrospective analysis of statin use in ovarian cancer [50, 51], endometrial cancer [51], gliomas [52], colorectal cancer [53], hepatocellular cancer [54], and prostate cancer [55] reveal reduced overall mortality, suggesting that statin therapy may have a protective effect against some cancers. Studies have shown that statins can affect multiple stages of ovarian cancer progression including: metastasis [56, 57], chemoresistance [58], and cytotoxicity [31, 32, 59, 60]. Treating ovarian cancer cells with statins and inhibitors of RhoA resulted in decreased cell dissemination in a NUDE mouse model of ovarian cancer [57]. It is noteworthy that co-treatment with lipophilic statins and cisplatin or doxorubicin results in apoptosis through activation of the intrinsic and extrinsic apoptotic pathways [31, 32, 58–60]. All of these reported mechanisms may explain the anti-tumor effect of simvastatin in our mouse model of EOC.

Interestingly, we have discovered that the ovarian cancer cell reprogramming that occurs within the tumor microenvironment may involve the acquisition of a p53 mutation. Our data, and data from others, demonstrate that ID8 cells are p53 wildtype [61]. However, 28-2 tumor cells within abdominal ascites showed increased p53 levels, which is an indication of p53 mutation affecting protein stability [62]. These cells showed strong reactivity to an antibody specific to mutant p53 and sequencing data showed mutation in codon 273 of exon 8 within the DNA binding domain of p53. This R273H mutation in 28-2 cells is the most frequent *TP53* mutation in high-grade serous ovarian cancer and accounts for 8% of all *TP53* mutations [63]. This is classified as an oncomorphic, or gain-of-function, mutation associated with increased invasion, survival, proliferation, angiogenesis, and resistance to chemotherapy [64–66]. Interestingly, discordance between the p53 status of primary tumor cells and ascites-derived ovarian cancer cells has been reported in women where primary tumor cells are p53 wild-type, while ascites-derived cells have acquired a p53 mutation [67]. The factors involved in driving this mutation are currently unknown. Some hypothesize that the growth-factor- and hormone-rich environment within the ovary can increase the aggressiveness of transformed ovarian cancer cells, leading to upregulated metabolism, the formation of reactive oxygen species, and the induction of DNA damage [4]. Recent studies have implicated the fallopian tube epithelium (FTE) as the origin of high-grade serous adenocarcinoma. Mutations of TP53 are typically seen early in the FTE and some have suggested that follicular fluid released at ovulation can flow into the fallopian tube and can induce DNA damage and p53 mutation [68]. P53 mutations in the ovarian surface epithelial cells have also been implicated in the progression to Type II, high-grade ovarian carcinomas [69]. The spectral karyotype confirmed the origin of 28-2 from ID8 cells, as a similar pattern of numerical and structural abnormalities were detected as well as several 28-2 unique characteristics. The ploidy level of several chromosomes was reduced, and a few increased in 28-2 resulting in a 6.2% smaller genome size by flow cytometry. The increased frequency of abnormal centromeres in 28-2 could also contribute to the prominent numerical instability that was described in other highly amplified human ovarian tumor genomes [70]. However, the three unique reciprocal translocations and three deletions emphasize the activity of structural rearrangements during the development and adaptation of 28-2. Interestingly, the hypothesized breakpoints in T(4;8) and T(10;15) translocations might be connected to frequently observed breakpoints in human ovarian tumors [71], thus emphasize the potential functional role of these chromosome segments. Tumor cell genomic instability has been shown to be an important predictor of outcome in patients with ovarian cancer [72], and this may account for some of the aggressiveness seen in the metastatic cells within ascites. Mutant p53 is known to interact with other transcription factors and modulate expression and function of their target genes [19]. For example, mutant p53 interacts with the transcription factors SREBP-2 and nuclear factor Y (NF-Y) [13, 20–23]; both SREBP-2 and NF-Y can potently induce HMGCR expression, which catalyzes activation of the mevalonate pathway [24–26]. Indeed, we demonstrate via ChIP analysis that the reprogrammed 28-2 cells exhibited increased histone acetylation [K9, 14] of the NF-Y site in the *HMGCR* promoter region in the reprogrammed 28-2 cells (Figure 12), suggesting epigenetic upregulation of HMGCR transcription and ultimately activation of the mevalonate pathway in these cells. Activation of the mevalonate pathway by SREBP-2 has previously demonstrated to regulate localization and activation of YAP and TAZ, which are mediators of the Hippo pathway and are potent oncogenes [27, 28]. Similarly, NF-Y is known to increase expression of the Rho family of small GTPases [29]. Mutant p53 has been shown to increase synthesis of mevalonate, which drives a more tumorigenic phenotype in a 3D model of breast cancer [13]. Increased expression of mevalonate and sterol biosynthesis enzymes in this model was due to an association between mutant p53 and SREBP transcription factors. Although only in two murine and human cell lines, the ascites-derived cells with a p53 mutation demonstrate increased activity of the mevalonate pathway, as well as an increased susceptibility to stain-induced cell death, suggesting that in response to p53 mutation, there is an enhanced reliance on the mevalonate pathway for viability.

**Figure 13:**
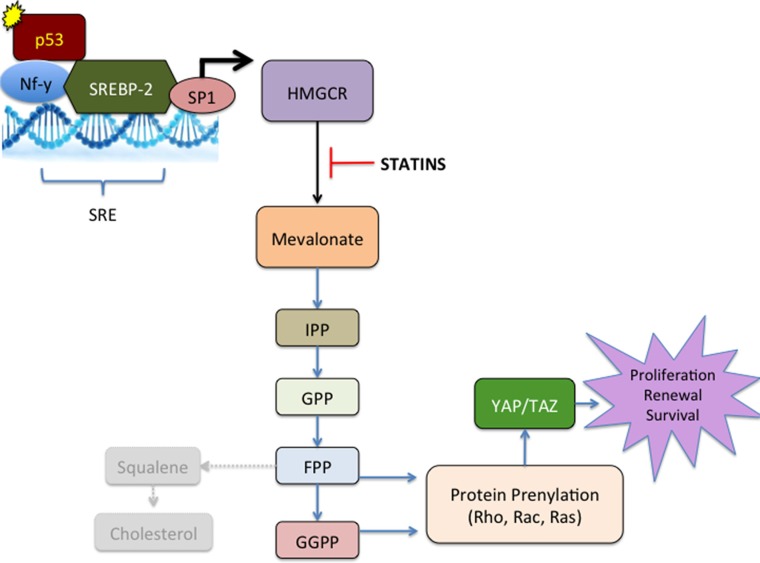
Working model of the reprogramming of metastatic ascites cells in ovarian cancer Following interaction with the ovarian tumor environment, circulating tumor cells acquire a p53 Mutation. Mutant p53 binds and activates transcription factors such as NF-Y and SREBP2 which bind to the sterol regulatory element (SRE) in the HMGCR promoter. Along with specificity protein 1 (SP1), NF-Y and SREBP2 increase transcription of HMGCR, which upregulates the Mevalonate pathway. Increased Mevalonate stimulates the formation of isopentenyl pyrophosphate (IPP). IPP can then lead to the formation of geranyl pyrophosphate (GPP) and farnesyl pyrophosphate (FPP). FPP can lead to synthesis of cholesterol through squalene and can also lead to prenylation of RAS. Our data suggest that cholesterol is not involved in the reprogramming of metastatic ascites cells. Geranylgeranyl pyrophosphate (GGPP) is responsible for prenylation of the Rho family of small GTP-binding proteins and subsequent activation of Hippo pathway proteins YAP and TAZ. Statin treatment blocks the enzyme HMG-CoA reductase (HMGCR) and its ability to produce mevalonate, effectively inhibit downstream process of this pathway.

With this enhanced reliance on the mevalonate pathway, targeting this pathway with statins can preferentially and potently induce death of tumor cells within the abdomen. As the metastatic abdominal tumors appear to have a similar upregulation of the mevalonate pathway seen in the ascites-derived cells, we hypothesize that the abdominal tumors may also be exquisitely sensitive to statin inhibition. Our *in vivo* experiments of advanced stage disease support this hypothesis, as indicators of disease morbidity were dramatically reduced following simvastatin treatment started in mice with advanced stage ovarian cancer. This is a significant finding, as the abdominal disease associated with advanced stage ovarian cancer is most often responsible for the morbidity and mortality associated with this disease. Our working hypothesis is that mutant p53 induces NF-Y and SREBP-2 (and possibly others) to transcriptionally activate HMGCR, resulting in upregulation of the mevalonate pathway, enhanced GTPase activity, and stimulation of survival pathways, such as the HIPPO pathway (Figure 13). Our study also demonstrated significant genomic instability in the re-programmed ascites derived 28-2 cells, which may have also contributed to the increased aggressiveness and reduced apoptosis evident in these cells. Increased copy number variations has been implicated in the development of aggressive high grade serous EOC [73, 74], and may lead to genetic influences in addition to the p53-mediated changes seen in our cells.

In summary, this study illustrates that mouse tumor cells exposed to the ovarian microenvironment acquire gene mutation and experience genomic instability, which is associated with increased mevalonate pathway activity. Due to the reliance on the mevalonate pathway, simvastatin is particularly cytotoxic to reprogrammed, ascites-derived murine and human ovarian cancer cells. Our *in vivo* data provide pre-clinical evidence to support continued exploration of statin use in advanced stage human EOC.

## MATERIALS AND METHODS

### Reagents and cell lines

Simvastatin, geranylgeranyl pyrophosphate (GGPP), farnesyl pyrophosphate (FPP), mevalonate (MEV), squalene and water-soluble cholesterol were purchased from Sigma. Simvastatin was prepared according to Sadghi et al., 2000 to activate the drug into its proform [75]. GGPP, FPP, MEV, squalene and water-soluble cholesterol were prepared according to manufacturer's instructions. Culture medium, glutamine and fetal bovine serum (FBS) were purchased from Life Technologies. The following spontaneously transformed mouse ovarian surface epithelial cells (MOSEC) lines were cultured with DMEMwith 10% FBS: ID8, 28-2, 30-2, IP and OT. The 28-2, 30-2, IP and OT lines were derived from ID8 cells that were obtained from mice after grafting ID8 cells into various microenvironments: 28-2 and 30-2 were isolated from the ascites of intrabursal injected cells and had thus interacted with the ovarian microenvironment. IP cells were obtained from the ascites of following intraperitoneal injection and had interacted with the peritoneal, but not the ovarian environment. The OT line was obtained from explant culture of ovarian tumors from an intrabursal injection, and thus had interaction with the ovarian microenvironment, but had not circulated within the peritoneum [33]. Human EOC lines, OVCAR(ATCC) and SKOV3 (ATCC) were cultured in RPMI with 20% FBS and McCoy's 3A media with 10% FBS respectively. Primary human EOC cells (iOvCa130 and iOvCa147 cell lines) were cell lines derived from the ascites of patients with Stage IIIC high-grade serous ovarian cancer patients and cultured in DMEM with 10% FBS and 1% antibiotic/antimycotic (Gibco). All human cell lines were collected and handled in accordance with ethics approval and patient consent guidelines at Western University (HSREB 12668E).

### RNA extraction and hybridization to genechip arrays

Total RNA was extracted from 1 × 10^7^ MOSEC cells grown on 100 mm culture dishes using RNeasy Minikits (Qiagen). RNA was extracted from triplicate values of ID8, 28-2 and IP and OT cell lines, quantified using a UV-VIS spectrometer (Nanodrop ND-1000) and RNA integrity was confirmed using the Agilent 2100 Bioanalyzer RNA Nano kit LabChip kit (Agilent Technologies). A total of 2 μg of total RNA was labelled using Agilent's Low RNA Input Amplification kit for 2-colour design for hybridizing to the Whole Mouse Genome Array (Agilent).

### Statistical analysis of gene expression profiles

Quality control of expression data was confirmed using the ARRAYQUALITY package in R and Bioconductor [76]. Data was preprocessed and normalized using Agilent's spatial detrending normalization and imported into GeneSpring for analysis. Data was log (Base 2) transformed, median centered, and divided into three groups: ID8, 28-2, and IP. Prior to analysis, the data was filtered to remove the confounding effect probes that show no signal may have on subsequent analysis [77]. Only probes that were in the upper 20 to 100th percentile of the distribution of intensities in 100% of any of the 1 of 3 above categories were allowed to pass through this filtering. A one-way ANOVA using a FDR Benjamini and Hochberg multiple testing correction (*q* < .05) [78] was performed, followed by a Tukey HSD *post-hoc* test for differences between select groups. Overlapping sets of significant probes were generated to find line specific lists. For example, to generate a 28-2 specific gene list, shared probes between 28-2 vs ID8 and 28-2 vs IP were determined. These results were further filtered with a minimum 2-fold difference cut-off. Gene ontology analysis [79] was used to determine functionally significant sets of co-regulated transcripts (*q* < 0.1 cut-off). All clustering was done using two-way hierarchical clustering with a Pearson centered distance metric and average linkage rules.

### WST1 viability assay

To determine the effects of simvastatin treatment on cell lines tested, cells were plated in 96-well plates, incubated for 48 hrs before serum deprivation and treated 24 hrs later with various doses (0.01, 0.05, 0.1, 0.5, 1.0, 5.0, 10, 20, 50 and 100 μM) of simvastatin. A second treatment was performed 48 hrs post-initial treatment and cells were incubated with the tetrazolium salt, WST1, according to manufacturer's instructions (Roche). After incubation at 37°C for 2 hrs, plates were read on spectrometer at 450 nm with a reference wavelength of 630 nm on an EL800 Universal microplate reader (Biotek Instruments, Inc). For rescue experiments, cells were co-incubated with 20 μM simvastatin and either 500 μM mevalonate, 10 μM squalene, 1 μM cholesterol, 5 μM GGPP or 50 μM FPP at the zero time point and 48-hr time point followed by data collected by WST1 as described above.

### Immunofluorescence

Immunofluorescence was performed on *in vitro* cultured ovarian cancer cells to determine expression of p53 and mutant p53, and changes in localization of SREBP-2, RhoA, YAP, and TAZ following simvastatin treatment. Briefly, for determination of p53 status, ascites-derived human ovarian cancer cells were cultured on glass coverslips in DMEM containing 10% FBS until 60–80% confluent. Cells were then fixed with 10% neutral-buffered formalin and permeabilized in 1% Triton X-100. Cells were incubated with anti-total p53 (Abcam ab26; 1:600 dilution) or anti-mutant p53 (Abcam ab32049; 1:500 dilution) overnight at 4^°^C. Cells were then incubated with secondary antibody (anti-rabbit 1:100 dilution) for 2 hours at RT, washed and counterstained with DAPI before mounting on glass slides with Prolong Gold antifade (Cell Signaling). Expression of SREBP-2, RhoA, YAP, and TAZ were localized in murine (ID8, 28-2) and human (iOvCa130, iOvCa147) ovarian cancer cells that either remained untreated, or were treated with 20 μM simvastatin for 24 hr. Cells were fixed and permeabilized as above and then incubated with SREBP-2 (Abcam ab28482 1:200 dilution), RhoA (Abcam ab32046; 1:400 dilution), YAP (Santa Cruz sc-101199; 1:100 dilution), or TAZ (Santa Cruz sc-48805; 1:200 dilution) antibodies overnight at 4^°^C. Fluorescence-conjugated secondary antibodies (Invitrogen, 1:100 dilution) were then added for 2 hrs at room temperature, and cells were mounted as above. Images were captured with an Olympus epi-fluorescence microscope and integrated morphometry software (MetaMorph; Burlingame, CA).

### TUNEL assay

MOSEC lines were cultured on glass coverslips in 24-well plates for 48 hrs before serum starvation overnight, which was followed by 0.1, 1.0, 10.0 and 50 μM simvastatin treatment. A second treatment at 48 hrs was followed by 10% formalin fixation at 60 hrs and storage in PBS at 4°C. To fluorescently label apoptotic cells, the terminal uridine nucleotide end-labeling (TUNEL) reaction was performed on sections to identify DNA strands breaks, a hallmark of apoptosis using the *in situ* cell death detection kit (Roche). Briefly, cells were permeabilized with Triton X-100, incubated with TUNEL reaction mixture, counterstained with DAPI and mounted with prolong gold (Life Tech). Photomicrographs were collected using an Olympus BX61 fluorescent microscope at 200× magnification and percent TUNEL-positive cells were determined using metamorph version 7.6.0.0 (Molecular Devices) from 5 fields of view. All TUNEL experiments were repeated in triplicate. Add-back experiments were conducted in a similar fashion except cells were co-treated with 20 μM simvastatin and 500 μM mevalonate, 5 μM GGPP or 50 μM FPP.

### Scratch assay

ID8 cells were seeded in 6-well plates in 10% FBS growth medium. After serum deprivation for 24 hrs, cells were treated with sublethal doses (0.5, 1.0 and 3.0 μM) of simvastatin. After 48 hrs, cells were wounded by scratching the plates with 200 μl pipette tip, washed with PBS and media containing simvastatin. Three areas were marked along the scratch and photomicrographs were collected at 4× magnification with an Olympus 1 × 71 inverted microscope. Additional micrographs were collected at the indicated areas 48 hrs later and the area migrated was measured using image J software. The percent wound closure was calculated by dividing the distance at time zero from the distance of the wound at the final time point.

### ChIP assay

ChIP was performed using a modification of previously published methods [80, 81]. ID8 and 28-2 cells at 70–80% confluency (3 – 1 × 10^7^ cells/per cell line) were washed once with PBS and incubated with 1% formaldehyde (in control medium) for 10 min at room temperature to cross-link proteins and DNA. Precleared chromatin was aliquoted into 300 μl amounts and incubated with 4 μg antibodies against acetylated histone H3 [K9,14] (Cat #05-399 from Millipore) at 4°C overnight. Two aliquots were reserved as controls – one incubated without antibody and the other with non-immune IgG. Protein A/G Plus agarose beads (60 μl) were added to each tube, the mixtures incubated for 2 h at 4°C and the immune complexes collected by centrifugation. Real-time PCR was employed using forward (5′-AAGTTCAGAGAGGCCATGAAGGGA-3) and reverse (5′-TCTGCAGTTATTAACCCAGCCGGT-3′) primers that amplify a ~100 bp region surrounding the proximal NF-Y response element, of the mouse *HMGR* promoter [24]. This region is conserved between human, mouse, and rat in the *HMGR* promoter [24]. Using serial dilutions of human chromosomal DNA, these primers were demonstrated to have equal efficiency in priming their target sequences.

### DNA sequencing

RNA was extracted from ID8 and 28-2 cell lines with RNeasy plus mini kit (Qiagen #74134) After PBS wash, 1 × 10^6^ cells were collected in the lysis buffer and following the manufacturer's instructions. cDNA synthesis was performed using 1 ug RNA with Quanta qScript cDNA supermix (Quanta #95048-100), according the manufacturer's protocol. Primers were designed with Geneious Pro 3.8.5 software on mouse *Trp53* cDNA (GenBank cDNA isoform a gi|187960038|ref|NM_011640.3|). One of the primer pairs annealed to exon-exon junctions to avoid amplification of genomic DNA. Primer sequences were as follows:

mp53cdna-F1: GCTCACCCTGGCTAAAGTTC; mp53cdna-F2: CAAGTCTGTTATGTGCACGTACTCTC; mp53cdna-F3: AGGGAGCGCAAAGAGAGC;

mp53cdna-R1: TGAGGGGAGGAGAGTACGTG; mp53cdna-R2: GTGGGCAGCGCTCTCTTT; mp53c dna-R3: AAAAGAGGGAGACAGGGTGG. Oligos were ordered from Laboratory Services Division University of Guelph. PCR products were amplified using 1 ul cDNA in 20 ul reactions contain 10 ul AmpliTaqGold 360 master mix (Applied Biosystem #439881) and 2 ul 5 pM forward-reverse primer mix. PCR products were confirmed with electrophoresis on 1% Agarose gel. PCR samples were sent for purification and Sanger sequencing to the Genomics Facility, University of Guelph. Sequencing data was analyzed and assembled to the mouse *Trp53* gene cDNA reference (GenBank cDNA isoform a gi|187960038|ref|NM_011640.3|) with Geneious Pro 3.8.5 software.

### Chromosome analysis by flow cytometry and spectral karyotyping

For spectral karyotyping, metaphase chromosome spreads were prepared from ID8 and 28-2 cell cultures grown in DMEM/10% FBS/1% ABAM/2% L-Glutamine medium by adding 0.04 μg/ml KaryoMax Colcemid for 1hr and followed by trypsin, hypotonic and MeOH/AcOH 3:1 fixative treatments according to standard cytogenetic procedures. Slides were aged overnight at 37°C in a dry oven prior to spectral karyotyping, according to manufacturer's recommendations (Applied Spectral Imaging). Briefly, slides were treated with pepsin, dehydrated in ethanol series, denatured at 72°C for 1 min, then quenched in ice-cold ethanol series. Probe mixtures were denatured at 80°C for 7 min and pre-annealed at 37°C then applied to the slides and hybridized at 37°C overnight. Washes were performed in 0.4% SSC at 72°C for 5 min and in 4 × SSC/0.1% Tween 20 at room temperature, followed by sequential blocking steps of incubation at 37°C for 30 min, thendetection reagents at 37°C for 45 min and triple washes in 4 × SSC/0.1% Tween 20 at 45°C for 2 min each. Finally DAPI/anti-fading reagent was applied to the slides and imaging was done using the SkyView system (Applied Spectral Imaging). The total nuclear DNA content was measured by a BD FacsScan flow cytometer using a standard protocol including ethanol fixed, RNase treated and propidium iodide stained cells from ID8, 28-2 and a normal fibroblast control. The data was analyzed by an univariate cell cycle model and the mean G1 peak values were compared among cell cultures in FlowJo V10.1.

### Immunoblot and cell fractionation

Cells and tumors were lysed and subjected to Western blot analysis as we have done previously [82, 83]. Membranes were probed overnight at 4°C for total p53 (Abcam ab26; 1:500 dilution), mutant p53 (Abcam ab32049; 1:500 dilution), Yap (Santa Cruz sc-101199; 1:500 dilution), Taz (Santa Cruz sc-48805; 1:500 dilution), HMGCR, (Santa Cruz sc-271595; 1:1000 dilution), Rac1 (Santa Cruz sc-95; 1:200 dilution), or RhoA (Abcam ab32046; 1:500 dilution). Membranes were then incubated with anti-rabbit or anti–mouse secondary antibodies (Sigma; 1:5000 dilution). Western Lightning Chemiluminescence Reagent Plus (PerkinElmer BioSignal, Inc., Montreal, QC). Computer assisted densitometry was performed using AlphaEase FC software (AlphaInnotech, San Leandro, CA) and results were quantified and reported as integrated densitometry values (IDV) relative to β-actin or GAPDH. For cell fractionation, cell cytoplasmic and nuclear fractions were separated according to the REAP protocol [84]. Briefly, ID8 or 28-2 cells were cultured in SF media, or were treated with simvastatin (20 μM) for 24 hr and scraped in ice-cold PBS. Cells were triturated in 0.1% Igepal (ca-630; Sigma) in PBS and centrifuged. The supernatant represented the cytoplasmic fraction. The remaining pellet was resuspended in 0.1% Igepal and re-centrifuged to generate the nuclear fraction. Samples were boiled in sample buffer and prepared for SDS PAGE and analyzed by Western blot analysis as above.

### Animal trials

C57Bl6 mice were purchased at Charles Rivers Labs and housed at the Central Animal Facility, University of Guelph, under the guidelines for Canadian Council for Animal Care. ID8 cells were injected into the ovarian bursa of recipient mice as described before [33]. Briefly, mice were anesthetised with isofluorane, a small midline dorsal incision was made and ID8 cells (1.0 × 10^6^ cells in 5 μl of PBS) were injected into the left ovarian bursa with a Hamilton syringe. After incision closure with staples, the mice were monitored as they recovered for the next 24 hrs. To determine the effects of simvastatin treatment on ovarian cancer progression, mice received daily intraperitoneal injection of 1 mg/kg/day simvastatin or PBS as vehicle control one week after the bursal injection of the ID8 cells (*n* = 6 mice/group). At 60 d post tumor induction, mice were euthanized by CO_2_ asphyxiation and ovarian weight was quantified. To determine the effect of simvastatin treatment on advanced-stage disease, tumors were induced as above and were allowed to grow without intervention for 60 d, at which time mice exhibited large primary tumors, numerous metastatic abdominal tumors, and abdominal ascites. Mice were then treated with daily intraperitoneal injections of 1 mg/kg/day simvastatin or PBS for 20 d until 80 d post-tumor induction (PTI) (*n* = 6 mice/group). At 80 d PTI, mice were euthanized and tumors removed and weighed, the number of metastatic abdominal tumors were counted to generate a scoring system (0 peritoneal tumors – 0; 1–3 peritoneal tumors − +; 4–10 peritoneal tumors − ++, and 11 or more peritoneal tumors − +++) and ascites-derived cell viability was assessed using a Trypan Blue exclusion test. Some of the metastatic abdominal tumors were also removed for histological analysis of members of the mevalonate pathway. To compare expression and localization of members of the mevalonate pathway, a portion of primary ovarian, and metastatic abdominal tumors were fixed overnight in 10% neutral buffered formalin, processed, and embedded in paraffin wax and cut into 5 μM sections on glass coverslips. Paraffin-embedded sections were deparafinized, rehydrated and endogenous peroxidases were blocked by incubation with 1% hydrogen peroxide for 10 min at room temperature (RT). Antigen retrieval was conducted by immersing sections in 10 mM citrate buffer at 90°C for 12 minutes. Tissues were blocked for 10 min at RT with 5% normal serum and incubated with anti-HMGCR (Santa Cruz sc-271595; 1:200 dilution), Rac1 (Santa Cruz sc-95 ; 1:400 dilution), or RhoA (Abcam ab32046; 1:400 dilution) overnight at 4°C in a humidity chamber. Slides were incubated with biotinylated secondary antibody (Sigma-Aldrich Canada Ltd., Oakville, ON) for 2 hours at RT followed by ExtrAvidin (Sigma-Aldrich; 1:100 dilution) for 1 hour at RT. The primary antibodies were visualized using DAB (Sigma-Aldrich Canada Ltd., Oakville, ON) and counterstained with Carazzi's Hematoxylin. After slides were dehydrated and mounted on coverslips they were imaged using brightfield microscopy. Another portion of the primary and metastatic secondary tumors was lysed in RIPA buffer and subjected to Western blot analysis as described above.

### Statistics

Data values are presented as the mean ± standard error (SE). Significance and *p*-values were determined with ANOVA followed by Tukey's post hoc test. For ChIP experiments to quantify acetylation, *t*-test was used. Differences were considered when *p* values were < 0.05. All experiments were independently performed at a minimum of three times.
